# Editorial: Efficacy, safety and biomarkers of novel therapeutics and regimens in the peri-operative setting of bone and soft tissue sarcoma

**DOI:** 10.3389/fsurg.2023.1228469

**Published:** 2023-08-09

**Authors:** Qiyuan Bao

**Affiliations:** ^1^ Department of Orthopaedics, Ruijin Hospital, Shanghai Jiao Tong University School of Medicine, Shanghai, China; ^2^ Shanghai Key Laboratory for Bone and Joint Diseases, Shanghai Institute of Traumatology and Orthopedics, Shanghai, China

**Keywords:** sarcoma, osteosarcoma, neoadjuvant, biomarker, peri-operative

**Editorial on the Research Topic** Efficacy, safety and biomarkers of novel therapeutics and regimens in the peri-operative setting of bone and soft tissue sarcoma

Recent strides in the field of oncology have witnessed new advances and hope for patients with bone and soft tissue sarcomas. This research topic explores the evolving landscape of novel therapeutics and regimens in the peri-operative setting, highlighting their efficacy, safety, and the role of biomarkers in guiding treatment decisions for treating these rare diseases.

The paradigm shift from amputation to limb salvage surgery seen 3 decades ago reflects the emergence of peri-operative chemotherapy and the understanding of tumor biology of sarcoma by sarcoma surgeons and tremendously impacts the treatment regimens of sarcoma (1). Similarly, the dawn of precision medicine and the emergence of targeted therapies has kindled a flicker of optimism, illuminating new possibilities in the fight against these complex cancers (2).

In this research topic, four exciting studies are focusing on various aspects of biomarkers and multimodal therapies for bone and soft tissue sarcoma, including a machine learning algorithm to predict the metastasis of osteosarcoma (Li et al.), the early experience of implementing cytoreductive surgery (CRS) plus hyperthermic intraperitoneal chemotherapy (HIPEC) for peritoneal malignancies (Zhu et al.), the safety and risk factors of chest wall malignant tumor as well as the clinicopathological features of giant cell hemangioblastoma (Li et al.). These results have greatly improved our therapeutic armamentarium against bone and soft tissue sarcoma in the peri-operative stage.

While these studies provide novel insights to the landscape of sarcoma management, further research encompassing genetic alterations, gene expression patterns, and immune signatures is needed to be incorporated into the treatment regimens and the selection of appropriate therapies at the molecular level. Advancements in genomic sequencing, transcriptomics, and proteomics have facilitated the identification of potential biomarkers, paving the way for the development of such personalized treatment approaches. For example, preoperative therapies, including neoadjuvant chemo-radiation (3) and other novel modalities, aim to optimize surgical outcomes by reducing tumor size and enhancing resectability. Ongoing studies strive to determine the most effective sequencing, combination, and duration of these peri-operative interventions, balancing efficacy with the minimization of potential toxicities (3).

While progress in sarcoma research fuels optimism, challenges remain on the path toward widespread implementation of these innovations. Combining targeted therapies into the peri-operative setting poses a threat of increasing post-surgery complications (4). Additionally, the identification of artificial intelligence predictive algorithms needs to be further standardized in its methodologies and validated in clinical trials (5). The most reasonable combination of multimodal approaches for sarcoma is still an ongoing area of exploration (2).

In conclusion, recent advancements in the efficacy, safety, and biomarkers of novel therapeutics and treatment regimens have infused the peri-operative management of bone and soft tissue sarcomas. The journey toward personalized medicine, guided by the intricate interplay of oncology practice and translational research, offers new avenues for improved patient outcomes. Continued collaboration among clinicians, researchers, and industry stakeholders is essential to surmount these challenges.
